# The ‘migratory connectivity’ concept, and its applicability to insect migrants

**DOI:** 10.1186/s40462-020-00235-5

**Published:** 2020-12-04

**Authors:** Boya Gao, Johanna Hedlund, Don R. Reynolds, Baoping Zhai, Gao Hu, Jason W. Chapman

**Affiliations:** 1 Department of Entomology, Nanjing Agricultural University, Nanjing, China; 2 Centre for Ecology and Conservation, University of Exeter, Penryn, Cornwall UK; 3 Lund University, Department of Biology, Centre for Animal Movement Research, Ecology Building, SE-223 62 Lund, Sweden; 4 Natural Resources Institute, University of Greenwich, Chatham, Kent UK; 5 Rothamsted Research, Harpenden, Hertfordshire UK; 6 Environment and Sustainability Institute, University of Exeter, Penryn, Cornwall UK

**Keywords:** Bogong moth, Brown planthopper, Compass orientation, Fall armyworm moth, Green darner dragonfly, Monarch butterfly, Windborne migration

## Abstract

Migratory connectivity describes the degree of linkage between different parts of an animal’s migratory range due to the movement trajectories of individuals. High connectivity occurs when individuals from one particular part of the migratory range move almost exclusively to another localized part of the migratory range with little mixing with individuals from other regions. Conversely, low migratory connectivity describes the situation where individuals spread over a wide area during migration and experience a large degree of mixing with individuals from elsewhere. The migratory connectivity concept is frequently applied to vertebrate migrants (especially birds), and it is highly relevant to conservation and management of populations. However, it is rarely employed in the insect migration literature, largely because much less is known about the migration circuits of most migratory insects than is known about birds. In this review, we discuss the applicability of the migratory connectivity concept to long-range insect migrations. In contrast to birds, insect migration circuits typically comprise multigenerational movements of geographically unstructured (non-discrete) populations between broad latitudinal zones. Also, compared to the faster-flying birds, the lower degree of control over movement directions would also tend to reduce connectivity in many insect migrants. Nonetheless, after taking account of these differences, we argue that the migratory connectivity framework can still be applied to insects, and we go on to consider postulated levels of connectivity in some of the most intensively studied insect migrants. We conclude that a greater understanding of insect migratory connectivity would be of value for conserving threatened species and managing pests.

## Background

‘Migratory connectivity’, a concept that is widely used in the vertebrate (particularly avian) migration literature, describes the extent to which different parts of a species’ annual range are linked by the movement paths of individuals [1–3]. Migration systems where a large proportion of individuals from the same breeding area migrate to the same non-breeding area along the same routes, with little mixing with individuals from other regions, are described as having relatively high (or strong) connectivity [4, 5]. By contrast, relatively low (or weak) connectivity occurs where individuals from one discrete breeding (or wintering) region separate during migration and spread between two or more regions, mixing with individuals from other breeding (or winter) regions [3–5]. Cohen et al. (2018) provide a clear comparison of different levels of migratory connectivity in three North American bird migrants, ranging from complete connectivity between breeding and wintering populations of rose-breasted grosbeaks (*Pheucticus ludovicianus*), intermediate connectivity (with full mixing on either side of a natural geological divide) in cedar waxwings (*Bombycilla cedrorum*), and no connectivity (complete mixing of all breeding populations in a single wintering area) in green-winged teals (*Anas carolinensis*) (see Fig. 1 in [4]). The degree of migratory connectivity has clear implications for vertebrate species conservation and management, because adverse environmental change at a specific location is expected to impinge on species with high connectivity more seriously than those with low levels [3, 6, 7]. However, it is still a challenge to quantify the exact degree of connectivity between breeding and wintering ranges [4, 5], even for vertebrates large enough to carry individual tracking devices (let alone insects), and there is an urgent need to make quantitative comparisons of migratory connectivity between species in order to understand the impact of migration on annual population dynamics, conservation status, and environmental policy [4].

**Fig. 1:**
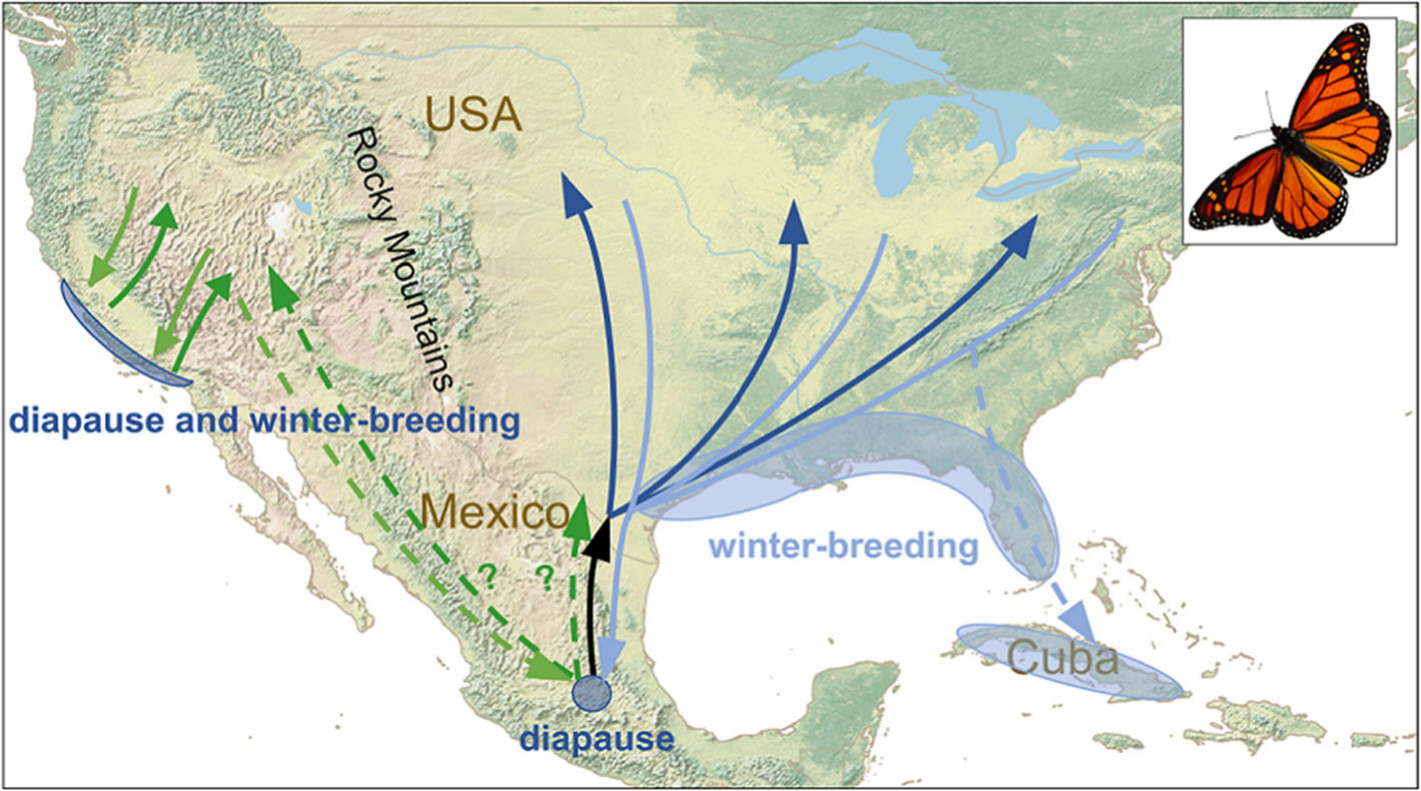
The annual migration circuit of monarch butterflies (*Danaus plexippus*) in North America. In the eastern population, the cycle starts in spring with migration of post-hibernation adults to Texas (black arrow), where they breed and die. The progeny then colonise the rest of the eastern North American summer-breeding range over the course of an additional 1–3 generations (dark blue arrows). During the autumn, most monarchs that have emerged in eastern North America migrate back to the central Mexican hibernation site (light blue arrows). However, the situation is complicated by an increasing trend for winter migrants to join the continuous-breeding, non-migratory populations in Florida, Cuba and along the Gulf Coast (dashed light blue arrow). The western population was traditionally believed to winter exclusively in hibernation sites along the Californian coast, involving a two-way migration (dark and light green arrows), but there is also an increasing trend for winter breeding at the coastal sites. However, recent evidence indicates that some western monarchs migrate to the central Mexican wintering ground in autumn (dashed pale green arrow), where they will mix with eastern monarchs. It is not known whether these western monarchs migrate back to the western or eastern summer-breeding range (dashed dark green arrows), but it is clear that population mixing is higher, and migratory connectivity is lower, than was originally believed

The concept of migratory connectivity is much less frequently applied to insects, with the exception of the monarch butterfly (*Danaus plexippus*) in North America [8–12]. Here we consider how, and to what extent, the connectivity framework is applicable to a variety of *long-range* insect migrants that undertake population movements traversing hundreds or even thousands of kilometers during their annual cycles (i.e., over spatial scales similar to those considered in the context of vertebrate migratory connectivity). However, if we wish to apply the migratory connectivity paradigm to insects, we need to consider three respects in which insect migrations differ in important ways to bird migrations, which we discuss in turn below.

Firstly, vertebrate migrants typically live for multiple years, while insect migrants nearly always complete a generation in considerably less than a year, often living for just a few weeks in the migratory (adult) stage. Accordingly, in the great majority of insect species, migration circuits are multigenerational, typically involving a minimum of three, and often six or more, discrete migratory legs, with each stage of the journey carried out by a new generation of adults [9, 13–16]. There are a few exceptions to the multigenerational pattern that we discuss below, but they constitute a small minority of migrant species. Migratory connectivity describes the spatial and temporal linkages of individuals and populations between seasons that result from migratory movements, and in vertebrates this usually entails regular seasonal migration from a breeding site to a nonbreeding site and back by the *same* generation of individuals. To make the concept applicable to most insects, however, we need to consider all the successive generations that contribute to the annual migration circuit (i.e. the ‘population trajectory’ through space and time, see [17]). In many insect migration systems, the population trajectory will have a tangled reticular form [18], rather than anything approaching a simple transition between specific sites.

Secondly, in the bird literature, migratory connectivity is usually discussed in terms of the degree of mixing, on the wintering grounds or along the migratory routes, of individuals from discrete breeding populations (sometimes separated by regions of unsuitable habitat or barriers [19]). By contrast, many insect migrants have annual, to-and-fro movements between extensive latitudinal zones (e.g., between northern temperate Europe and the Mediterranean Basin), without any clear form of geographical structuring or discrete populations across their broad range [13, 14, 20, 21]. In bird connectivity terms, these may be regarded as having very low or no connectivity, because they may be considered as a single, completely mixed, population. However, within these broad zones of summer-breeding and winter-breeding habitat, there may still be a degree of connectivity, for example if individuals that developed in the western part of the summer-breeding range migrate exclusively or principally to the western part of the winter-breeding range, and vice versa (as probably occurs in the painted lady butterfly, *Vanessa cardui* [22]). Thus, if we extend the definition of migratory connectivity to consider the degree of linkage within sections of a continuous and extensive range, then insect migratory circuits can be encompassed within the connectivity framework.

Thirdly high-flying insect migrants, due to their comparatively slow self-powered flight speeds in relation to typical wind speeds [23], will tend to have a lower degree of control over their movement directions than faster-flying birds [24], and consequently they may be expected to have much lower connectivity. Some large and comparatively powerful day-flying migrants, for example butterflies and dragonflies, circumvent this issue by largely restricting flight activity near to the ground where they can exert a greater degree of control over their movement direction [14]. Such ‘flight boundary layer’ migrants [25, 26] are the exception among insects however, where migration at high altitude is the norm [14]. Thus it is important to understand that terms such as ‘high’ and ‘low’ connectivity are relative and need to be defined with respect to the taxa under consideration. For example, insect migratory circuits defined as involving comparatively high connectivity may not be directly comparable, in absolute terms, to birds that have been similarly characterised.

Taking these three aspects of insect migration into consideration, we believe that the migratory connectivity framework can be applied to insects. Here we review what we know about annual circuits, migration routes and migratory connectivity in insects, and highlight the (admittedly huge) areas of ignorance on this topic. In the following sections, we examine degrees of connectivity in insect migratory circuits, using examples with well-characterised migration routes to illustrate the different levels of connectivity within two broad categories of migrant. Firstly, we discuss migrants that exert a significant, but varying, degree of influence over their movement pathways (‘directed migrations’); such movements are expected to show comparatively high degrees of connectivity. Secondly, we focus on migrants which have little or no influence on their movement directions, relying almost entirely on ambient winds for transport (‘non-directed migrations’); such movements are expected to show low, or no, connectivity.

### Directed migrations

#### Butterflies (Lepidoptera)

The ability to maintain a constant and relatively unvarying movement trajectory over long distances is an attribute typically associated with butterfly migrations [14, 26]. Thus we may expect butterflies to show the highest levels of migratory connectivity among insects, especially in species with geographically separated populations. This high level of directedness arises from the interaction between two components of butterflies’ movement ecology: sensory physiology and flight behaviour. Tethered-flight experiments under controlled conditions, and vanishing bearing measurements of free-flying butterflies, have shown that migrant butterflies consistently take-up preferred flight headings in adaptive, seasonally-reversed, directions [26–29]. Butterflies generally use a time-compensated solar compass to select a migratory direction [27, 30–32], but it is far more challenging to maintain this bearing over long distances under natural conditions, when the migrants will be exposed to varying (and often unfavourable) wind conditions. Butterflies are generally considered to overcome this problem by migrating within the flight boundary layer, where winds are slower than their self-powered flight speed [23, 25]. Such flight behaviour enables migrating butterflies to compensate for crosswind drift experienced en route, allowing them to precisely control the direction of their movement trajectory [23, 26]. However, recent evidence indicates that butterflies often fly high above the ground when winds are at least partially favourable in order to increase their displacement speed [13, 14, 20], which inevitably will have consequences for the directedness of their movements. Further work is required to investigate the regularity and impact of high-altitude flights on the migratory routes and destinations of butterflies, and for the majority of species the degree of connectivity between parts of the annual range is simply not known.

The one species of butterfly for which we have a good understanding of the level of connectivity is the monarch, at least in its North American range. Traditionally, North American monarchs are assumed to comprise two geographically separated populations (Fig. 1). The population to the east of the Rocky Mountains breeds in northeast USA and southeast Canada during late summer. Their progeny enter reproductive diapause and migrate up to 2500 km to the southwest each autumn, to hibernate in forests in the mountains of Michoacán, central Mexico [9, 31, 33]. The following spring the butterflies emerge from hibernation, break reproductive diapause, and mate, before migrating to the Texas/Louisiana area where they produce the next generation. The progeny of these spring breeders then colonise the breeding range over the course of an additional 1–3 generations [8, 9]. Monarchs breeding to the west of the Rocky Mountains have a similar annual cycle, but were (until recently) assumed to hibernate exclusively on the southern Californian coast [33–35]; this scenario has recently required some qualification, as described in detail below. Monarchs exert a high degree of control over their movement directions: for example, eastern monarchs tethered in a ‘virtual flight simulator’ [27] during the migration season consistently head southwest in the autumn but northeast in the spring [27, 31, 36]. These flight directions are highly consistent with the expected migratory route between Mexico and northeast USA (Fig. 1), which is achieved by recourse to a complex sensory system [31, 33] but a relatively simple set of navigational rules [30]. Given the highly unusual situation of, (i) apparently discrete eastern and western breeding populations (separated by the Rocky Mountains), (ii) highly localised hibernation sites seemingly exclusive to each population, and (iii) a great degree of control over their migratory directions, monarchs were expected to show high connectivity [9, 12]. Due to considerable concern over large-scale declines to the migratory populations in both the east and west [10, 37] of the range, knowledge of the level of migratory connectivity is important in terms of future conservation strategies.

A number of recent observations have greatly complicated the picture, however, and indicate that the degree of separation between the two populations is not as complete as traditionally assumed (Fig. 1). A model of western monarch migration routes, based on the location of collected specimens, confirms that all monarchs breeding in the Pacific coastal states (California, Oregon and Washington) winter in California as expected, but suggests that some monarchs breeding in inland western states (Idaho and Montana) migrate along riverine corridors through Nevada, Utah and Arizona towards Mexico during autumn [34]. The model receives support from frequent observations of autumn migrants in Utah, Nevada and Arizona exhibiting flight headings towards the south and southeast [38], consistent with migration to Mexico. These inland western monarchs appear to show intermediate migratory behaviour, as individuals tagged in Arizona have been relocated at either the central Mexican or the Californian hibernation sites [35]. Mating between western and eastern monarchs in central Mexico will lead to a degree of mixing via return spring migration of eastern females to Texas that are carrying mixed progeny. Whether return migration of monarchs from Mexico to the western breeding grounds occurs remains an open question, due to a lack of direct observations of migration along this potential route. However, the strong correlation between the size of the winter population in Mexico and the number of monarchs in California the following summer [39] indicates that migration by this route may well be substantial. These recent observations of a much greater degree of mixing between eastern and western monarchs in Mexico suggest that migratory connectivity will be lower than previously believed. Indeed, molecular studies indicate that all North American monarchs constitute a single panmictic population that shows a lack of genetic divergence between eastern and western monarchs [40, 41].

The migratory system in the east is further complicated, and undergoing rapid change, by a recent trend for alternative wintering destinations and loss of migration, and it is becoming clear that a substantial number of eastern monarchs no longer reach Mexico (Fig. 1). A significant proportion of eastern monarchs now migrate via Florida in the autumn, where they break reproductive diapause and become subsumed within the resident, continuously breeding, populations in south Florida [11] and Cuba [42]. Additionally, an increasing proportion of autumn migrants break reproductive diapause and abandon migration in the Gulf Coast region of Texas, to join the small, but increasing, resident breeding population in this region [43]. This change is induced by recent widespread planting of tropical milkweed (*Asclepias curassavica*) in gardens, an exotic larval food-plant which has enabled winter-breeding to take place, and it appears that migration is easily lost in monarchs [36]. Thus, the switch of many migrants to winter breeding in southern Florida and Texas leads to the loss of individuals from the eastern migratory population and a consequent reduction in migratory connectivity, and may also be one of the drivers of the long-term decline in the size of the Mexican winter population [10, 12, 37].

What is clear from this brief overview of the recent literature is that the North American monarch migratory system is far more complex than previously believed, and is also highly dynamic with a recent trend towards the loss of migratory activity. The monarch is the most intensively studied insect migrant by far, but despite the vast number of published studies, we still don’t fully understand the level of migratory connectivity, nor the implications of the changing wintering ecology for the population dynamics and conservation status of this iconic species. It is also clear that research into the migration routes, population dynamics, and levels of connectivity are urgently needed for the hundreds of other migratory butterfly species, for which we often lack even the merest notion of where they persist at certain times of the year.

#### Noctuid moths (Lepidoptera: Noctuidae)

To-and-fro migrations between climatic zones are widespread among moths in the family Noctuidae [14]. Migratory noctuids (including genera such as *Agrotis*, *Euxoa*, *Helicoverpa*, *Heliothis*, *Mythimna*, *Noctua* and *Spodoptera*) comprise some of the world’s most serious agricultural pests, colloquially referred to as cutworms, armyworms, bollworms, budworms and earworms, and they are prevalent throughout Eurasia, Africa, Australia and the Americas [14, 20, 44–47]. Many species use high-altitude winds to rapidly move between extensive latitudinal zones, and, as indicated by simulated migratory routes [48, 49], most species will likely experience a high degree of mixing resulting in low connectivity. Here we discuss two comparatively well-studied noctuids where the evidence indicates they have a higher degree of connectivity than the norm, due to the occurrence of highly localised ranges during a part of the annual cycle.

**Fig. 2:**
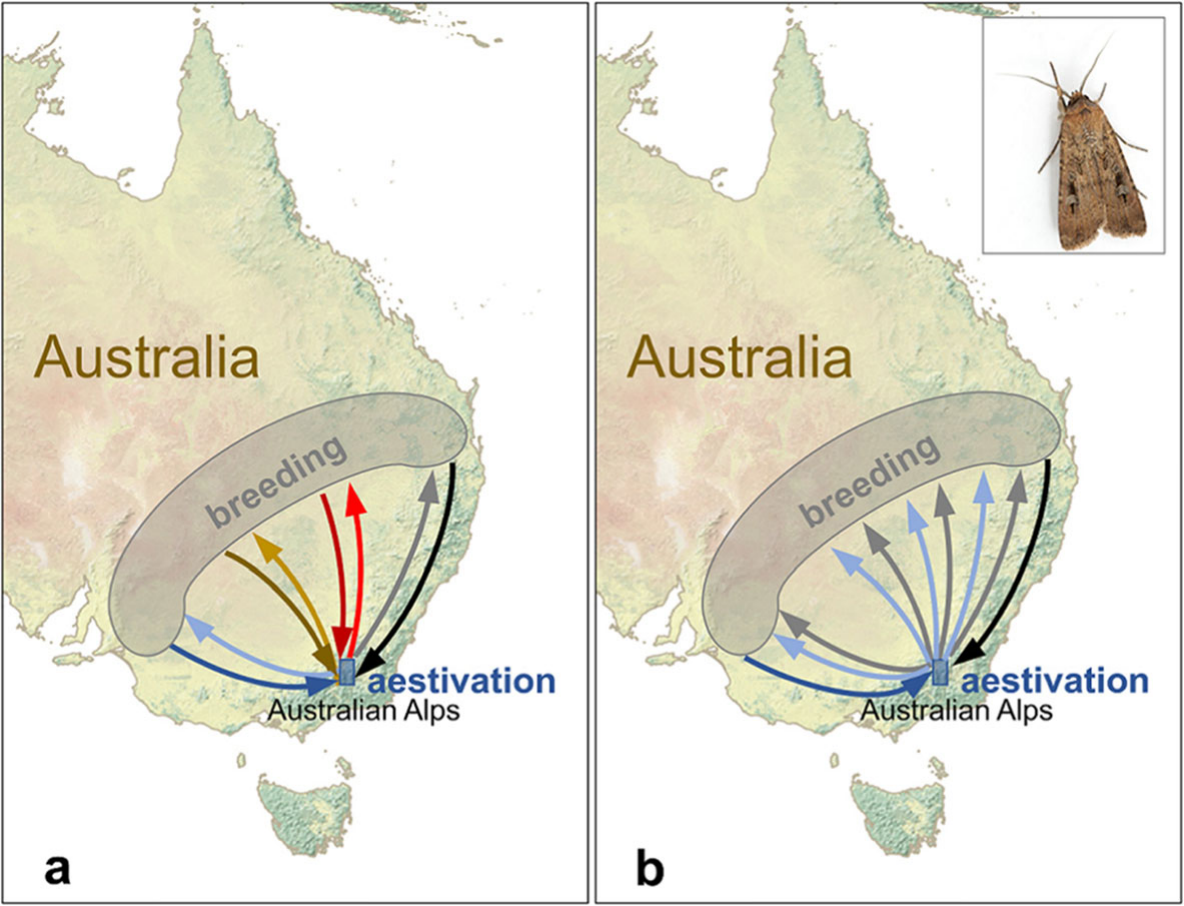
The migratory circuit of the Bogong moth (*Agrotis infusa*). Bogong moths breed during the southern winter in semi-arid regions of inland south-east Australia (grey shaded area). In spring, adult moths migrate from this region (dark coloured arrows) to the highly localised alpine caves in the Australian Alps, where they spend the hot, dry summer in torpor (aestivation). In the autumn, the same adults migrate back to the breeding range (light coloured arrows), where the univoltine breeding cycle commences again. The level of connectivity in this migration cycle is not currently clear. **a** One possibility is that the level of connectivity is very high, with moths originally from the south-western part of the breeding range returning there after winter hibernation (blue arrows), and moths from the north-eastern part of the range returning there (black and grey arrows). **b** Alternatively, connectivity may be very low or non-existent if moths originally from one particular part of the breeding range (say the south-western portion, dark blue arrow) return to all parts of the breeding range after hibernation (light blue arrows)

The quintessential example here is the Bogong moth (*Agrotis infusa*), an iconic migrant inhabiting southeast Australia [46] (Fig. 2). Migratory populations occur to the west of the Great Dividing Range, in the dry inland plains of southern Queensland, western New South Wales (NSW) and western Victoria, where larvae develop on a range of broad-leaved herbaceous plants (including vegetable crops) over the southern winter. In spring the newly emerged adults migrate up to 1000 km south or east to the Australian Alps, to escape the hot and dry summer conditions of the lowland plains. Here they congregate in the tens of thousands in more than 40 cool alpine caves and crevices in mountain ranges straddling the Australian Capital Territory, southeast NSW and northeast Victoria (Fig. 2), where they enter a period of torpor known as aestivation [46]. In the autumn the adults awake from their dormant state, and migrate north or west back to the breeding grounds, where they mate, lay eggs and die, thus starting the annual cycle once again.

There is clearly a degree of connectivity in the Bogong’s migration cycle, as all the migratory populations breeding across an extensive swathe of inland southeast Australia (Fig. 2) congregate annually in a comparatively small mountainous area to the south or east of the breeding range [46]. The navigational mechanisms by which Bogong moths locate the geographically restricted alpine caves is an area of active research [46, 50]; it appears they use a combination of a magnetic sense and visual landmarks to take up headings consistent with navigation from the breeding plains to the mountains each spring, and back again the following autumn [50]. However, the strength of the connectivity in this migration system is not currently understood. For example, it is plausible that migratory connectivity could be very high: if moths from a particular part of the breeding range (say, the north) migrate to just a small subset of the caves used, and these moths are genetically programmed to *reverse* their incoming migration direction when they leave, they will return to their original emergence site with little mixing with moths from other locations (Fig. 2b). Alternatively, moths originating from any particular region of the breeding range may spread out across all the suitable aestivation caves, and/or return to any part of the breeding range after aestivation, resulting in a high degree of mixing with moths from all other breeding areas, and consequently low or zero connectivity (Fig. 2a). Further work on the preferred migration directions and navigational capabilities [50] of moths from all parts of the breeding range is required to answer this question and resolve the level of connectivity in this iconic migration.

The Bogong moth has a mixed strategy of long-range migration and dormancy, similar to the monarch butterfly. However, it differs in several respects: the dormant period is over the summer rather than the winter; it migrates at night and thus navigation is more challenging than for diurnal migrants [46, 50]; and the annual migratory circuit is carried out by a single generation. Such single-generation (or univoltine) migrations are comparatively rare among insects; in moths, they seem to occur only in cases where the movement is between low-altitude winter-breeding regions and communal high-elevation summer aestivation sites, before migration back to the breeding area. The Siberian cutworm (*Euxoa sibirica*) in Japan [51], and the Jersey tiger moth (*Euplagia quadripunctaria*) on the Greek island of Rhodes [52], appear to be species with a similar strategy to the Bogong, spending the dry summer period aestivating at higher elevations than the breeding area. Army cutworm moths (*Euxoa auxiliaris*) also carry out long-distance migrations from their breeding grounds (the North American Great Plains) to high elevation sites (the Rocky Mountains), however the moths remain active rather than aestivating [53]. During this period they feed on rich nectar sources at night, markedly increasing their body mass and lipid content, and conceal themselves between the rocks of talus slopes during the day (in numbers large enough to provide a significant source of food for grizzly bears) [53]. In the same way as the Bogong moth, these species also have the potential to have high levels of connectivity in their migration circuits, but we know considerably less about their migratory patterns and navigational capabilities, and these species represent great opportunities for further study of the level of connectivity.

**Fig. 3:**
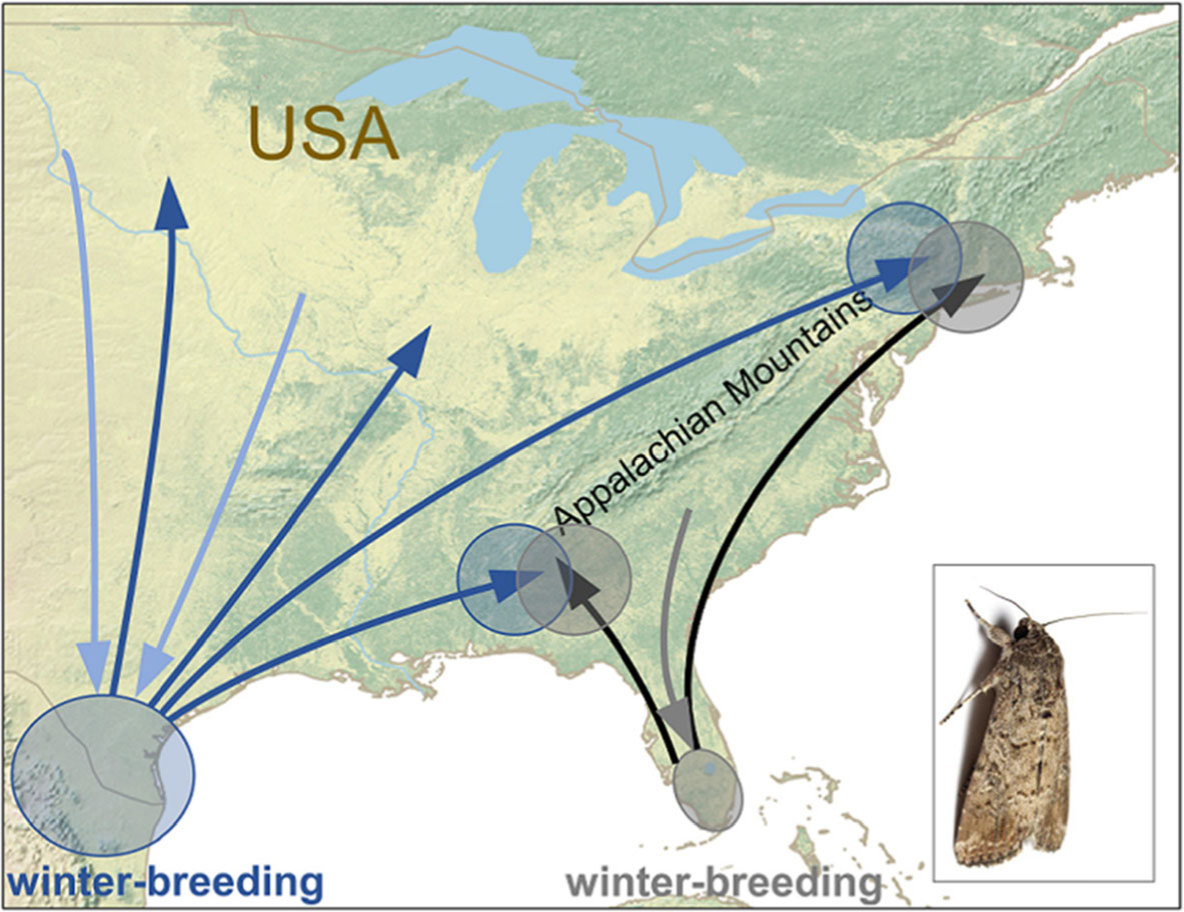
The annual migration circuit of fall armyworm moths (*Spodoptera frugiperda*) in eastern North America, an example of comparatively high connectivity. The populations of fall armyworm which breed in Texas (blue) and Florida (black and grey) during the winter can be reliably identified by their haplotype ratios, allowing the migratory pathways of these two populations to be delimited. The Texas population expands during the spring and summer over the course of several generations throughout eastern North America to the west of the Appalachian Mountains (dark blue arrows), and returns to Texas during the autumn (light blue arrows), largely without mixing with the Florida population. These moths expand into the region largely east of the Appalachians each spring (black arrows) and return to Florida in the autumn (grey arrow). There is only a limited amount of hybridisation between the Texas and Florida populations during the summer-breeding period, in the regions to the south and north of the Appalachians (overlapping blue and grey circles)

The fall armyworm (*Spodoptera frugiperda*) is a crop pest native to the New World that is incapable of diapause, and so unlike the species discussed so far, it breeds continuously [54] (Fig. 3). In North America, fall armyworm winter-breeding generations are restricted to latitudes below 28° N in southern parts of Texas and Florida, but each year, over the course of several generations, they expand up to 3000 km northwards to colonise the whole of the eastern USA and parts of southern Canada by late-summer [54–56]. During the autumn, some moths return south, via windborne transport on northerly winds associated with the passage of cold fronts [45, 57]. The migration pattern is therefore similar to that of the monarch in some respects, as both species seasonally expand over the entirety of the eastern USA by emigration from a large winter population southwest of the summer-breeding range (Texas and Mexico, respectively) and a smaller winter population southeast of the summer range (Florida; Figs. 1 and 3). In other respects the migration is very different. Fall armyworm carry out rapid windborne migrations hundreds of meters above the ground in just a few nights [47, 56], and thus their level of directional control will be relatively low. Monarchs on the other hand typically fly closer to the ground and therefore travel much slower, taking several weeks to complete a migratory leg, but consequently with much greater control over the direction of their movements.

Given the disparity in the levels of directional control, one would expect fall armyworm to experience higher mixing of migratory routes, and therefore to exhibit a lower degree of connectivity than monarchs. However, evidence from genetic monitoring of population structure in the eastern USA surprisingly indicates the contrary. Fall armyworm originating from Texas show reproducible differences in haplotype frequencies of the *Cytochrome Oxidase I* gene compared to fall armyworm from Florida [58], and this provides a method of testing the provenance of moths and delineating the migratory pathways throughout the summer range. The geographical distribution of Texas and Florida populations has been mapped throughout the eastern USA by molecular determination of field collections over multiple years, and this study indicated there is only a limited region of overlap and mixed breeding [55]. It appears that the Appalachian Mountains play an important role in segregating the migratory pathways, largely keeping the Texas and Florida populations separated along the potential region of overlap, with the exception of the southern and northern fringes of the mountain range where they come into contact and interbreed (Fig. 3). The persistence of the genetically distinct Texas and Florida lineages, despite migration into the same summer region providing an annual opportunity to mix, indicates that fall armyworm populations in North America have a surprisingly high degree of connectivity, despite the windborne nature of their migratory journeys [47, 56]. This pattern is in stark contrast to the monarch butterfly, which notwithstanding its seemingly greater directional control, appears to experience a greater degree of population mixing across its entire summer range (Fig. 1) [40, 41]. The level of detail known about the spatial population dynamics and migratory routes of the two noctuids described here is rather atypical for the family, and for most species we are unable to assess the level of migratory connectivity (albeit we predict it will be considerably lower or zero in most migrant noctuids); given the agricultural and economic significance of this group, this situation should be urgently addressed.

#### Dragonflies (Odonata)

Dragonflies are strong fliers and regular long-distance migrants [15, 59–63]. Dragonfly migration is often reported to occur in large swarms [59], a behaviour that facilitates detection by human observers, but accounts of single vagrant individuals are also numerous in the literature [59, 62]. Annual migration circuits in dragonflies are completed over the course of several generations [15, 61], in a similar manner to many other insect migrants. Considering how well-known and well-liked dragonflies are, it is astounding how little we actually know about their migratory systems [59, 62], and this lack of knowledge will necessarily limit an analysis of migratory connectivity in this group. Nevertheless, the fragmentary knowledge that exists suggests that dragonflies are as adept at migration as butterflies, having the ability to accomplish movements along a preferred flight trajectory [26, 64, 65], an important prerequisite of migratory connectivity. They typically achieve this by migrating close to the ground within their flight boundary layer, often against the wind [26, 60, 64], although at times they also engage in high-altitude windborne migration, especially when crossing water bodies [61, 66]. Open water crossings are most likely rare however, and migratory species have been shown to avoid long water crossings when possible [67]. Much research is still needed on the flyways, destinations and migratory behaviour of the World’s dragonflies before migratory connectivity can be assessed on a wider scale, but there is one species for which we have reasonably good information.

**Fig. 4:**
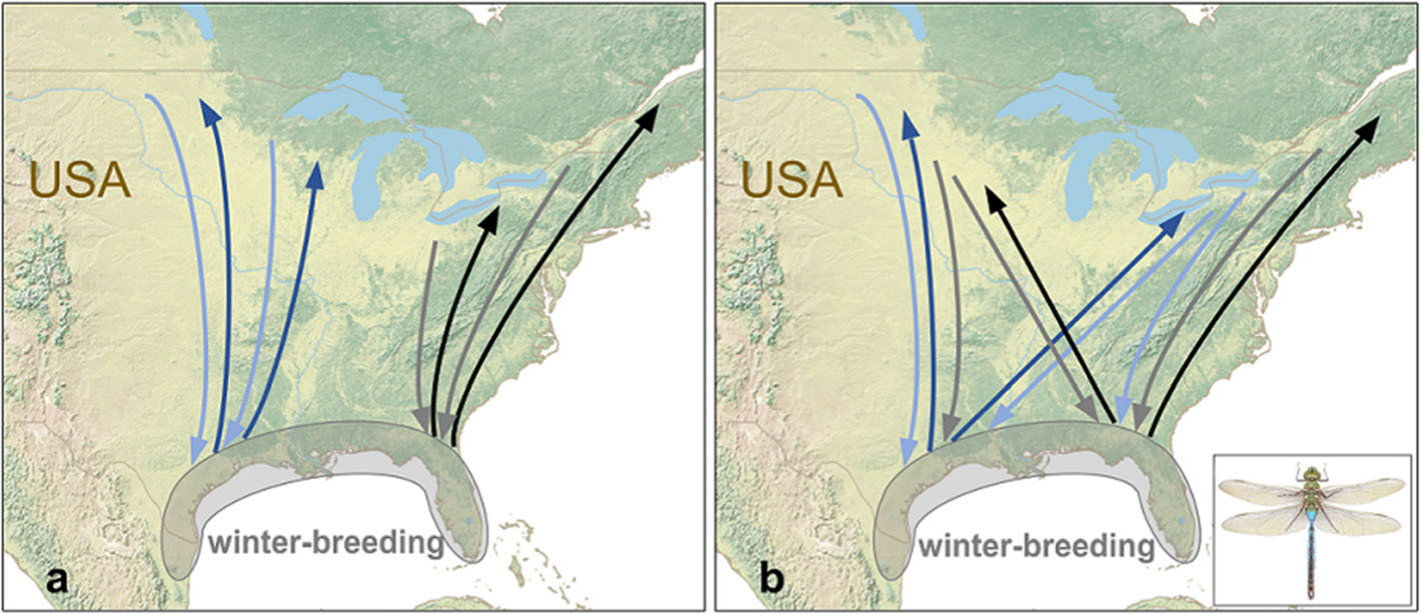
The annual migratory circuit of the green darner dragonfly (*Anax junius*) in eastern North America. Green darners have three generations per year: a spring migratory generation which travels from the southern coastal states as far north as southern Canada (dark blue and black arrows); an autumn migratory generation which returns to the southern coastal states (light blue and grey arrows); and a non-migratory generation which develops during the winter in the south (grey shaded area). The level of connectivity in this species is not clear. **a** One plausible scenario is that migratory connectivity is high, with populations west of the Appalachian Mountains (blue arrows) remaining largely separate from populations to the east (black and grey arrows). **b** An alternative scenario is that there is frequent mixing across the entire range, leading to a single panmictic population in eastern North America with low connectivity. Green darners are also found to the west of the Rocky Mountains, and it is not clear if this population is connected to that in the east. Further genetic and tracking studies are required to resolve these issues

The green darner (*Anax junius*) has the best-known migration of any dragonfly species [15, 59, 62]. The distribution includes North America, from southern Canada to Mexico and the Caribbean, with core areas appearing to be California, Texas, Florida and the eastern United States (Fig. 4). Sightings of large swarms of green darners have been reported continuously for decades and have elicited much interest and wonder [59, 62], but it was not until recently that the full scale of its complex, multi-generational, continent-wide migratory circuit was described in detail [15]. The generation that emerges in the southern part of the range, referred to as the first generation, migrates up to 600–700 km northwards over the course of the spring and summer to reproduce in the northern breeding range (Fig. 4). In May–July, the progeny of adults that arrived in the north in the previous summer emerge, forming the first cohort of a second generation. In September, green darner numbers peak in the north, as progeny of the earliest arriving migrants from the south emerge, forming a second cohort of the second generation, which immediately migrates south. The overwintering of late-stage nymphs in the north may have a latitudinal limit, determined by a temperature threshold [68]. Thus, at the species’ northern range limit, there may only be one cohort of second-generation green darners [69]. Arriving in the south in the autumn, migrants of the second generation reproduce and die, and their offspring, emerging in November, constitute a third, non-migratory generation. The progeny of this third generation gives rise to the individuals that migrate north the following spring [15].

In their study, Hallworth et al. [15] pooled data from geographically distant sample regions in the south (Texas, Florida, West Indies and Mexico), in order to provide evidence of a general northern origin of migrants arriving all across the south. From the data presented in Hallworth et al. [15], it is not possible to determine the extent of migratory connectivity occurring in this circuit. It is quite feasible, given the directional control exhibited by green darners [67], that eastern and western populations may remain separated (Fig. 4a), leading to high connectivity. By contrast, an earlier analysis of mitochondrial genetic markers, with sample sites ranging across North America, showed that the green darner lacks any obvious patterns of geographic structuring of haplotypes [63]. These data suggest that perhaps migratory connectivity is low, with individuals appearing to mix rather frequently across the entire continent (Fig. 4b). The genetic study did however discover that a surprisingly high degree of haplotype diversity has been maintained [63], despite the high level of gene flow, and this does indicate that some separation of populations or cohorts exists. In conclusion therefore, there are aspects of green darner migration that still require further investigation before we can completely understand the level of connectivity in the eastern North American population.

### Non-directed migrations

In contrast to the large, comparatively powerful, insect migrants discussed above, which are capable of determining or at least influencing their movement trajectory through their self-powered flight action, numerically the vast majority of insect migrants are small, weak-flying species that rely entirely on the wind for transport [70]. Among the best studied are pest Hemiptera, especially plant- and leafhoppers (suborder Auchenorrhyncha) and aphids (suborder Sternorrhyncha). The low self-propelled flight speeds (airspeeds) of small insects will make an insignificant contribution to their windborne ground speed, so there is no adaptive benefit in maintaining a specific flight orientation, either in relation to the wind or in a seasonally-preferred direction [23]. This is reflected in the observed orientations of small windborne migrants – these seem to be effectively random or, if common orientations *are* seen, they seem unrelated to the downwind direction or to assisting movement in any adaptive direction [71]. Small insects can, of course, exert some control over their general direction of movement by choosing when to fly, and there is one well-documented example, the potato leafhopper (*Empoasca fabae*; Hemiptera) in North America, that in autumn shows enhanced emigration in conditions favouring southward transport towards its overwintering areas [72]. Generally, however, the windborne movements of small insects seem to be initiated irrespective of wind direction as long as air temperatures are favourable for flight. Hu et al. [70], for example, concluded that the migration displacement directions of small insects (mostly aphids) in the UK corresponded to the prevailing wind directions, with no evidence of wind selectivity. The reason for this may be that the sought-after resources (e.g. patches of host plants) may lie in any geographical direction from the emigration site. The random directionality of movement may be useful in increasing population dispersal during migration, but does lead to a complete absence of a recognisable migratory circuit containing an element of ‘return’. A strategy of flight in winds from all directions will often entail high mortality, but the high mortality is compensated by the high fecundity and development rates of these species, which lead to high intrinsic rates of increase [73].

In some regions of the world however, particularly arid and semi-arid zones that experience a monsoon climate, seasonal wind patterns are directed in such a way that closed-loop to-and-fro migrations can evolve simply from a strategy of downwind transport lacking any form of wind selectivity during the initiation of migration. One such region is the savannah / Sahel zone of West Africa, where the progressive advance, in summer, of the Inter Tropical Convergence Zone is followed by a belt of intense convective rainfall in an otherwise arid zone. A wide range of windborne migrant insects move north into the Sahel on moist south-westerly monsoon winds in early summer, to take advantage of renewed growth of vegetation and other resources produced by the monsoon rains [74, 75]. Later (September–October) the Inter Tropical Convergence Zone retreats southwards again, and north-easterly ‘Harmattan’ winds are re-established which allow the progeny (or, in a few special cases, the original immigrants) to move south-westwards out of the increasingly dry Sahel. The utility of this atmospheric circulation is such that large numbers of species exploit it, most of them completely unstudied [75], but including tiny species such as mosquitos and other Diptera which are entirely windborne [75, 76] to large species such as grasshoppers and locusts (Orthoptera) that actively fly downwind [77]. Nothing is known about the degree of migratory connectivity in such populations; however, the windborne nature of the transport and lack of self-directed movement suggest that is it likely to be non-existent or at least extremely low.

**Fig. 5:**
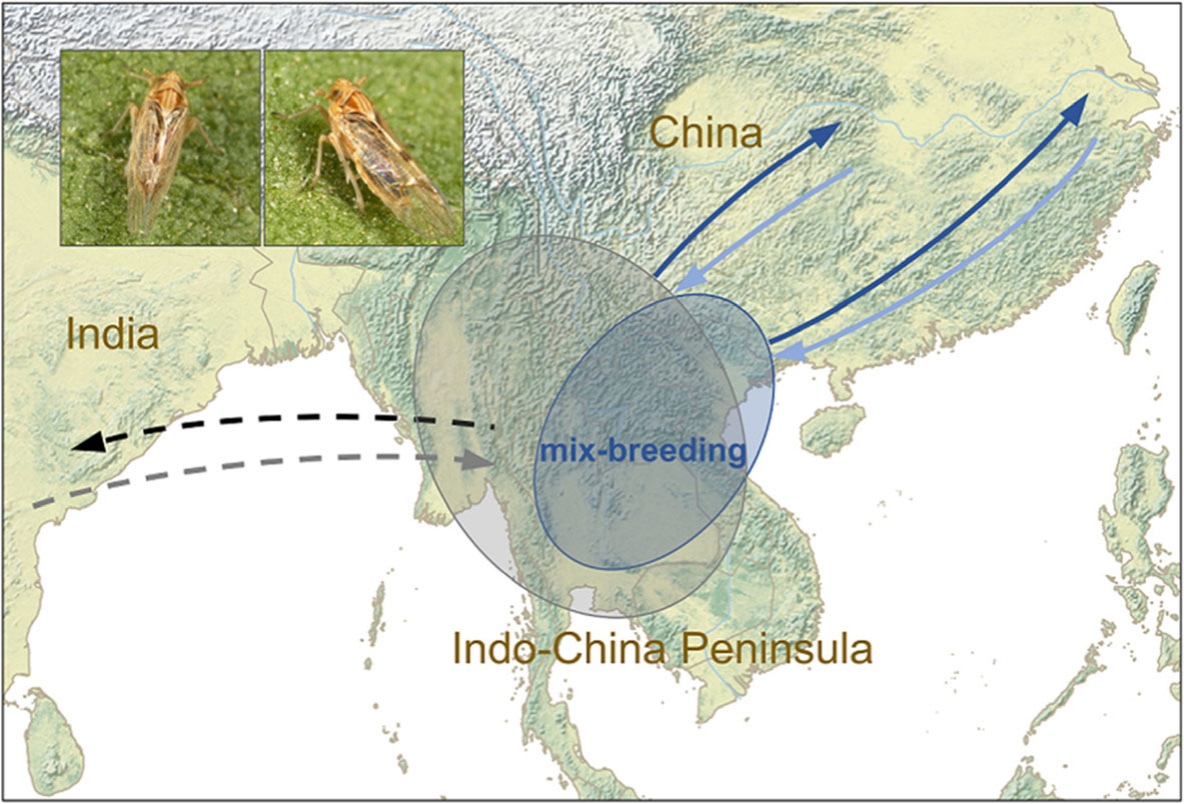
Mixing of brown planthopper (*Nilaparvata lugens*) populations in South Asia, South East Asia and East Asia. Brown planthoppers are resident breeders throughout tropical South and South East Asia. Each spring, brown planthoppers from the winter-breeding population in Indochina migrate north-eastwards to colonize East Asia (blue arrows), and it was generally considered that these made up a single South East / East Asian population which was distinct from the South Asian population, thus indicating a reasonably high level of migratory connectivity. However, a recent genetic analysis has indicated that a high degree of mixing occurs in northwest Indochina (overlapping blue and grey circles) due to regular exchange between here and South Asia (black and grey dashed arrows). Thus levels of migratory connectivity within the South Asian and South East / East Asian populations of brown planthoppers are lower than originally thought

An example of a windborne migrant in which we do know something about the degree of connectivity is the brown planthopper (*Nilaparvata lugens*; Hemiptera), a rice pest resident throughout South and South East Asia to northern Australia, and seasonally present in East Asia (Fig. 5). Each spring, migrations into temperate East Asia (China, Korea and Japan) from winter rice-growing regions in northern Indochina are facilitated by the prevailing south-westerly winds associated with the summer monsoon [16, 78]. In late summer and autumn, return migrations by later generations of brown planthopper are promoted by persistent north-easterly winds associated with the winter monsoon [71, 78]. This migratory loop between South East and East Asia is entirely regulated by the movement of the monsoon and associated winds [16], and it is generally assumed that this South East / East Asian migratory population is genetically distinct from the South Asian population [79]. This supposition is supported by phenotypic differences between brown planthopper from the two regions, such as distinct virulence levels against resistant rice strains [80]. A recent analysis indicates that the South East / East Asian and South Asian clades are somewhat genetically divergent, but that there is a greater degree of gene flow between the two groups than was previously suspected [79]. In particular, brown planthopper from Myanmar, Yunnan (southwest China), Thailand and Laos showed a high degree of ancestry from South Asia, but all samples from the South East / East Asian range had experienced some gene flow from South Asian populations [79]. These findings indicate that there must be frequent mixing between the South Asian and South East Asian populations, resulting in a relatively low level of migratory connectivity within these two geographic populations (Fig. 5).

## Conclusions

From the examples discussed in this review it seems evident that migratory connectivity in the sense used by vertebrate migration specialists has fairly limited applicability to insects. Most insect examples where we know enough about the population structure to draw conclusions indicate that migratory connectivity is low or absent, and there is widespread gene flow at the continental scale in the majority of species. As mentioned in the Introduction, the multigenerational nature of many insect migration circuits may reduce connectivity. In other words, it seems likely that if a *series of individuals*, a generation apart (all of which have rather weak control over their flightpaths) have to make decisions on migration flight timings and orientations through the course of a year, that will probably tend to increase the variability of migration landing areas, compared to a straightforward “out-and-back” seasonal migration made by the same individual (as occurs in many vertebrates). Moreover, the fact that insects rely on windborne transport to a far greater extent than birds, means that the lack of connectivity and large-scale mixing which is typically observed is not a great surprise. However, a few species such as the fall armyworm do show a surprisingly high degree of connectivity, and there are many other species for which a lack of empirical data preclude our ability to assess the level of connectivity.

For most insect species, the combination of high fecundity and probable weak migratory connectivity suggests that the degree of connectivity is unlikely to play a key role in population trends or conservation status. Bird fertility is generally low (clutch sizes ~ 1–10) compared to that of insects (100s–1000s) and mortality during migration (e.g. due to loss of habitat or hunting) along the route or at highly-localised stopover sites and overwintering areas may have very serious effects on a bird species, driving it towards extinction in some cases [81]. Thus the degree of migratory connectivity in bird populations is a key factor in their conservation [7]. Parallels exist in a few insect cases: for example, a reduction in good nectar resources for migrating monarchs, and anthropogenic interference with the overwintering aggregations, are both believed to play a role in the decline of this species in North America [37]. The migrant insect species most under threat tend to be those with highly specialised migration systems that combine migration with diapause at a localised site, such as the monarch. However, most migratory insects appear to not be threatened, combining high mobility with very high fecundity and thus they have the ability to withstand generational mortality of > 99% [48, 82]. These colonising, dispersive species with high rates of population growth can (and often do) bear high migration losses. As mentioned previously, the progression of a migrant population (usually composed of a series of temporary subpopulations) through its habitat areas in space and time will often have a complex reticular form [17, 18]. There will be many small ‘spurs’ on the connectivity reticulum signifying the destruction of certain subpopulations that have landed in unsuitable places, such as oceans [83, 84]. For example, there were colossal losses of desert locusts (*Schistocerca gregaria*) during the 1988 crossing of the Atlantic [83], where all the migrants died at sea or failed to breed successfully on arrival, but despite this there is no suggestion that the species is under threat. The concept of migratory connectivity therefore has little significance for insect conservation broadly (notwithstanding a few highly specialised species like the monarch).

On the other hand, elements of connectivity can be important for the management of migratory crop pests and disease vectors [16, 78]. Sometimes crops or livestock/human populations come under threat from migrant pests in *distant source areas* that are recognized from analogies with past situations (e.g. the complementary seasonal breeding areas, connected by swarm migrations, of the desert locust [85]). It may then be judicious to carry out control either in the source areas themselves, or during migration, before the pests reach the susceptible cropping areas or animal populations – so-called ‘preventive management’ [86]. Alternatively, warnings can be issued allowing ‘defensive’ control measures to be organised in the at-risk areas [16].

Further entomological radar [87], genetic [40, 41, 63, 79], stable isotope [11, 15, 28, 42] and individual marking [35, 88] studies carried out in other regions and biomes of the world are needed to answer questions about the level of connectivity in insect migration systems. An exciting recent development is the reduction in weight of electronic tagging devices [89] which may help to get better flightpath data for very large insects (such as green darner dragonflies [65, 67]), although it will take time to build up a database of migratory tracks (such as those emerging from decades of bird tagging).

## Data Availability

Not applicable.
